# Not all areas are alike: willingness to comply with pandemic containment measures across residential contexts in China

**DOI:** 10.3389/fpubh.2026.1911656

**Published:** 2026-09-07

**Authors:** Linxu Zhu

**Affiliations:** School of Political Science and Public Administration, Shandong University, Qingdao, China

**Keywords:** compliance willingness, pandemic containment measures, pandemic governance, public health policy, residential heterogeneity

## Abstract

**Introduction:**

Public compliance with containment measures is important for effective pandemic governance, but willingness to comply may vary across residential contexts. This study examined compliance willingness with COVID-19 containment measures across rural, town, and urban areas in China and the factors associated with it within each setting.

**Methods:**

Using cross-sectional survey data from 985 respondents across three northern provinces of China, we compared compliance willingness across residential contexts using nonparametric tests. We also estimated hierarchical block-entry OLS regression models for the three subgroups and conducted pooled residence-by-predictor interaction tests to assess contextual heterogeneity formally.

**Results:**

Compliance willingness differed significantly across settings. Rural respondents reported higher willingness than town and urban respondents, although the absolute differences were modest. The subgroup models showed different within-context patterns of association, with government performance positively associated with compliance willingness in all three settings. Formal interaction tests indicated that between-context heterogeneity was concentrated in the institutional domain and specifically in the association between institutional trust and compliance willingness.

**Conclusion:**

The findings reveal selective heterogeneity in compliance willingness across residential contexts, particularly in the role of institutional trust.

## 1 Introduction

Public compliance with containment measures is essential for effective pandemic governance and for limiting the spread of infectious diseases (1, 2). Previous health crises, including the H1N1 influenza pandemic and the West African Ebola epidemic, demonstrated the importance of public adherence to disease-control measures (3, 4). The COVID-19 pandemic brought this issue into particularly sharp focus on a global scale (5, 6). During the pandemic, governments worldwide implemented social distancing, mask mandates, restrictions on public gatherings, and other non-pharmaceutical interventions (7–9). These measures played an important role in slowing transmission, particularly during the early stages of the pandemic, before vaccines and effective treatments became widely available (10–13). Nevertheless, uneven compliance across populations reduced their effectiveness and created substantial challenges for pandemic control (14–17). Understanding the patterns and factors associated with public compliance therefore remains important not only for interpreting responses to COVID-19 but also for strengthening preparedness for future public health emergencies.

Public compliance with containment measures is associated with a range of psychological, institutional, social, and demographic factors (18–20). Protection Motivation Theory provides a useful, although partial, perspective for understanding the psychological component of compliance. It suggests that protective behavior is shaped partly by individuals’ appraisal of a threat and their assessment of possible coping responses (21). Consistent with this perspective, fear of contracting COVID-19 has been associated with behavioral changes, including adherence to social distancing and improved hand hygiene (22), while risk perception shapes how individuals assess the likelihood and potential consequences of infection (23, 24). However, pandemic compliance is not determined by threat appraisal alone. Institutional trust and evaluations of government performance may influence whether containment measures are perceived as credible and legitimate (25), while the behavior of community members, family, and friends provides social cues regarding appropriate conduct (26). Demographic characteristics may further shape exposure to risk, access to information, and responses to public policies (27). Accordingly, this study integrates the psychological, institutional, and social factors into three complementary mechanisms—threat appraisal, institutional evaluation, and social influence—whose relative importance may vary across residential contexts.

Although existing studies provide valuable insights into public compliance, much of the literature focuses on aggregate populations or individual-level differences, offering limited understanding of how willingness to comply varies across residential settings. This limitation is important because the mechanisms underlying compliance may be conditioned by the environments in which individuals live. Residential settings differ in their demographic, social, institutional, and public health characteristics, which may shape how residents perceive health risks, interact with public authorities, and respond to containment measures (28–31). Existing research, however, commonly conceptualizes these differences through a rural–urban dichotomy or continuum, which may obscure the intermediate and potentially distinctive position of towns as settings that share characteristics with both rural and urban areas (32–35). Consequently, limited attention has been paid to systematically comparing all three settings or examining whether the factors associated with willingness to comply vary among them.

China provides a relevant setting in which to investigate this residential heterogeneity. During the COVID-19 pandemic, containment policies were coordinated through centralized leadership and bureaucratic mobilization, while their local implementation relied substantially on local governments, grassroots administrative networks, and community-based organizations (36, 37). Within this broader institutional framework, residential settings in China vary in population density, public service provision, information access, community relationships, and local implementation conditions (38, 39). Comparing rural areas, towns, and urban areas within the same national context makes it possible to examine how local social and administrative environments are associated with willingness to comply while limiting some of the national-level institutional variation inherent in cross-country comparisons. Although China’s governance arrangements are context-specific, this comparison can offer broader insights into how residential conditions and local implementation environments shape public responses to health-emergency measures.

This residential heterogeneity also has practical implications. If the factors associated with willingness to comply vary across residential settings, a uniform public health strategy may generate uneven responses. Effective interventions may therefore require different combinations of risk communication, institutional trust-building, community engagement, and information provision across rural areas, towns, and urban areas. Identifying these context-specific patterns can help policymakers tailor public health interventions more closely to local conditions and make more informed decisions about the allocation of administrative and public health resources during future emergencies.

To address this research gap, this study examines public willingness to comply with COVID-19 containment measures across rural, town, and urban areas in China and identifies the factors associated with it within each setting. Drawing on cross-sectional survey data from 985 respondents in Beijing Municipality and Hebei and Liaoning provinces, the study uses comparative tests and grouped regression analyses to assess whether willingness to comply varies across residential settings and whether psychological, institutional, social, and demographic factors display different patterns of association across them. The study makes two principal contributions. First, it moves beyond the conventional rural–urban dichotomy by examining towns as a separate residential context rather than subsuming them within either rural or urban areas. Second, it examines residential heterogeneity not only in the level of willingness to comply but also in the associations of psychological, institutional, and social factors with compliance willingness.

## 2 Literature review and research hypotheses

### 2.1 Factors associated with compliance willingness

Existing studies examine public compliance through both adherence behavior and willingness to follow public health measures (40, 41). Evidence concerning compliance behavior therefore informs the present theoretical framework. In this study, however, the outcome refers specifically to respondents’ stated willingness to comply rather than observed behavior. Compliance willingness may be associated with psychological, institutional, social, and demographic factors (23, 24). Rather than operating as isolated sets of predictors, the psychological, institutional, and social factors represent three complementary mechanisms through which compliance willingness may be formed: threat appraisal, institutional evaluation, and social influence. Demographic characteristics provide background controls rather than a separate explanatory mechanism. This framework links the three domains through their common role in shaping compliance willingness while allowing their relative importance to depend on the informational, institutional, and social conditions of different residential contexts.

Protection Motivation Theory provides a useful, although partial, basis for understanding the psychological component of compliance. The theory proposes that protective responses are shaped by individuals’ appraisal of a threat and their assessment of available coping responses (21). In the context of infectious disease, fear can signal the perceived seriousness of a threat and motivate precautionary action. Research conducted during COVID-19 accordingly associates fear of infection with behaviors such as social distancing, mask-wearing, and improved hygiene practices (22). Risk perception is similarly relevant because individuals who consider infection both likely and consequential may be more willing to adopt preventive measures (42). These relationships are not unique to COVID-19: evidence from H1N1, Ebola, vaccination, and other preventive health contexts also identifies threat appraisal as an important correlate of protective behavior (4, 5, 42). Nevertheless, threat appraisal alone does not ensure compliance, because protective action also depends on whether individuals regard the recommended responses as effective and feasible. Its relevance may further vary with perceived threat severity and access to reliable information. Threat appraisal should therefore be understood as one component of compliance rather than as a sufficient explanation.

Institutional evaluation constitutes a second mechanism. Individuals may be more willing to comply when they regard containment measures as necessary, legitimate, and competently implemented (41). Trust in government and public institutions can facilitate acceptance by increasing confidence in the intentions, information, and capacity of authorities (43). However, studies do not report uniformly positive relationships. In some circumstances, high institutional trust may reduce precautionary behavior by creating confidence that the threat is already under control (44–46). These apparently conflicting findings may reflect differences in what trust represents and in the behaviors being examined. Confidence in the legitimacy and competence of public authorities may encourage adherence to official requirements, whereas confidence that authorities have successfully controlled the threat may reduce perceived personal risk and discretionary precautions. The relationship may also differ between mandatory compliance and voluntary protective behavior (41). Evaluations of government performance, institutional preparedness, and enforcement bodies are therefore relevant because they connect general trust to citizens’ more immediate assessments of how policies are implemented (41, 47). Institutional factors may consequently support compliance through both perceived legitimacy and confidence in implementation, but their relevance remains dependent on policy and crisis contexts.

Social influence provides a third mechanism. Individuals interpret public health guidance within communities and interpersonal networks rather than in isolation (48). The observed behavior of community members, family, and friends can provide descriptive information about what others do, while shared expectations communicate what others consider appropriate (23, 49). When compliance is visible and collectively supported, these social cues may reinforce behaviors such as mask-wearing and social distancing, particularly where group identification is strong (26). Social influence, however, is not uniformly beneficial. Excessive pressure may reduce voluntary cooperation or generate resistance, particularly when expectations are perceived as coercive or illegitimate (48). The consequences of social influence may therefore depend on whether community norms operate through shared identification and mutual support or through pressure and sanction. This distinction suggests that community and peer influence may operate differently in settings characterized by dense social relationships and those with more heterogeneous social networks.

Demographic characteristics are also associated with variation in compliance. Gender, political orientation, and related characteristics may affect exposure to health risks, access to information, and responses to public policy (7, 27). Unlike threat appraisal, institutional evaluation, and social influence, these characteristics do not constitute a single explanatory mechanism. Instead, they identify patterned differences among population groups and provide important background controls when assessing the psychological, institutional, and social correlates of willingness to comply.

Overall, the framework treats threat appraisal, institutional evaluation, and social influence as complementary mechanisms associated with compliance willingness, with demographic characteristics serving as background controls. Their relevance is conditional rather than universal: threat appraisal depends partly on information and perceived threat, institutional evaluation on policy credibility and implementation, and social influence on the structure of community relationships. These contextual contingencies provide the basis for examining how residential settings may condition the relative importance of the three mechanisms.

### 2.2 Residential contexts and compliance heterogeneity

Residential contexts differ substantially in their economic, social, and institutional characteristics, and these differences may shape the conditions under which public compliance develops. Urban areas generally have greater access to education, healthcare, and employment opportunities, whereas rural areas may face more persistent constraints in public service provision (28, 38). At the same time, rural and urban areas are interconnected and may exhibit different vulnerabilities that require context-sensitive rather than uniform policy responses (50). Differences in social organization, community relationships, and local living conditions further distinguish residential settings (51, 52). Residential context should therefore be understood not simply as a geographical classification, but as a configuration of informational conditions, institutional implementation environments, and social-relational structures. These conditions may shape how residents access and evaluate health information, interact with public authorities, and perceive the behavior and expectations of others in their communities.

The importance of contextual conditions is also evident in broader public health research. Studies have linked culturally grounded community knowledge to social cohesion and psychological resilience in rural and semi-rural China (53), environmental and energy conditions to population health across developing Asian countries (54), and educational and sanitary conditions to post-pandemic health risks among vulnerable groups in Romania (55). Although these studies examine outcomes other than compliance willingness, they reinforce the broader premise that health-related attitudes, responses, and vulnerabilities are embedded in local social, environmental, and institutional conditions.

More directly, residential differences are reflected in health-related attitudes, behaviors, and responses to public health risks. Rural and urban populations may differ in their value orientations and behavioral patterns (34). For example, research among cancer survivors has identified rural–urban differences in smoking, physical activity, and self-reported health even after individual characteristics were taken into account (29). During the COVID-19 pandemic, studies in some settings found lower uptake of mask-wearing and social distancing among rural residents (30, 31). Evidence from China further suggests that differences in information-evaluation capacity may contribute to rural–urban disparities in preventive behavior, highlighting the relevance of communication environments and cognitive barriers (39).

Towns require particular analytical attention. Bell’s account of the rural–urban continuum emphasizes that rural, town, and urban areas are connected through changing social, economic, and cultural relationships, while Dewey and Pahl caution against reducing community differences to population density or fixed territorial categories (32, 33, 35). This continuum perspective does not, however, imply that towns are merely a midpoint between rural and urban areas. Rather, towns may constitute a hybrid residential context in which relatively developed public services, formal institutional presence, and population mobility coexist with comparatively visible and cohesive community relationships (38, 51). This combination may expose residents simultaneously to formal institutional signals and community-based social norms (23, 49). Treating towns separately therefore makes it possible to examine a contextual configuration that could be obscured if they were subsumed within either rural or urban areas. Because the three settings represent different combinations of informational, institutional, and social-relational conditions, willingness to comply can reasonably be expected to vary across them. Accordingly, we propose:

*H1*: Public willingness to comply with pandemic containment measures differs across rural, town, and urban residential settings.

Residential context may also condition the relevance of the three mechanisms identified in Section 2.1. The residential categories are not assumed to determine compliance willingness directly; rather, they represent different configurations of informational, institutional, and social-relational conditions. First, differences in access to health information and the capacity to evaluate it may shape threat appraisal, including perceptions of the likelihood and severity of infection (39, 42, 56). Second, variation in local administrative capacity, public service provision, and interactions with authorities may shape institutional evaluation by influencing perceptions of policy credibility, government performance, and implementation effectiveness (41, 47). Third, the density and composition of community relationships may condition social influence, because norms and peer behavior may be more visible in closely connected communities but operate differently in settings characterized by greater mobility and social heterogeneity (23, 49). Towns may display a hybrid configuration in which formal institutional influence coexists with relatively visible community norms. Residential context may therefore be associated not only with different levels of willingness to comply but also with differences in the relative importance of these mechanisms. Because existing evidence does not support consistent directional predictions across rural, town, and urban settings, the hypotheses focus on contextual variation rather than specifying a predetermined direction. Accordingly, we propose:

*H2*: The associations of psychological, institutional, and social factors with compliance willingness vary across rural, town, and urban residential contexts.

**Figure 1:**
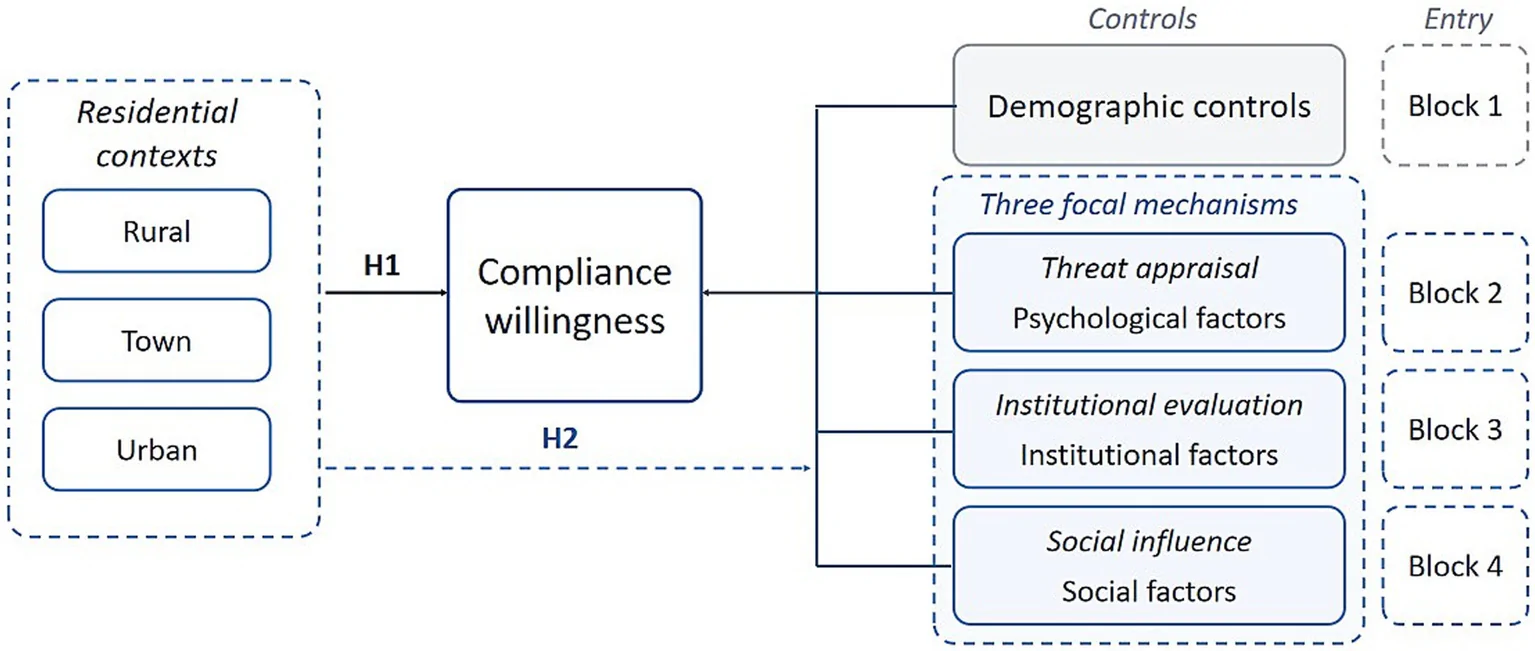
The conceptual model of the study.

The study’s conceptual model, presented in Figure 1, summarizes these proposed relationships and provides the analytical framework for the empirical analysis.

## 3 Materials and methods

### 3.1 Survey design

This study used data from a cross-sectional online survey conducted in northern China in November 2022 to examine public willingness to comply with COVID-19 containment measures and the factors associated with it. The questionnaire was administered in Chinese through the WJX online survey platform, and the survey link was distributed by research assistants to residents aged 18 years or older. Participation was voluntary, and the resulting dataset constituted a non-probability convenience sample. A total of 999 survey responses were initially collected. The survey platform restricted multiple submissions from the same IP address, and no duplicate submissions were identified. The average completion time was approximately 9 min (SD = 6.87). Submissions completed in less than 2.5 min or more than 30 min were excluded, resulting in a final analytical sample of 985 respondents.

The survey covered 26 cities across Beijing Municipality and Hebei and Liaoning provinces.^1^ Residential context was self-reported as urban, town, or rural, producing subsamples of 584, 313, and 88 respondents, respectively. No residential quotas or stratified sampling procedures were applied, and the unequal group sizes reflected voluntary participation. The use of non-probability online recruitment may have introduced self-selection and underrepresented individuals with limited internet access or digital literacy.

The questionnaire covered sociodemographic characteristics, psychological responses, institutional evaluations, social influences, and willingness to comply with containment measures. Before participation, respondents were informed of the study’s purpose, the voluntary nature of participation, and the anonymous collection of data. Electronic informed consent was obtained, no personally identifiable information was collected, and no financial incentive was provided.

### 3.2 Measures

The questionnaire items were developed for this survey to capture perceptions and behavioral intentions specific to the institutional and policy context of COVID-19 containment in China. The exact wording of all items is reported in Appendix Table A1.

#### 3.2.1 Dependent variable

The dependent variable was compliance willingness, measured using two complementary items. The first assessed respondents’ general willingness to follow COVID-19 containment regulations, while the second assessed their willingness to cooperate with police in the enforcement of those regulations. Both items were measured on a 5-point scale ranging from 1 (least willing) to 5 (most willing), and were averaged to form a composite measure, with higher values indicating greater willingness to comply. The two-item measure showed acceptable internal consistency (Cronbach’s *α* = 0.70). Although the two items refer to distinct forms of intended conduct, both concern respondents’ willingness to support the implementation of the same COVID-19 containment regime. The composite therefore captures stated willingness across general regulatory compliance and enforcement-related cooperation.

#### 3.2.2 Demographic variables

Five demographic variables were included to account for baseline sociodemographic variation: gender, age, educational attainment, marital status, and employment status. Gender was coded as 0 for male and 1 for female. Age was grouped into six categories: 18–21 (1), 22–29 (2), 30–39 (3), 40–49 (4), 50–59 (5), and 60 years or older (6). Educational attainment was classified as less than high school (1), high school diploma (2), vocational college (3), bachelor’s degree (4), and master’s degree or above (5). Marital status was classified as not currently married (0) or married (1), and employment status as not currently employed (0) or currently employed (1).

#### 3.2.3 Psychological factors

Three psychological variables were included: pandemic fear, perceived risk, and perceived stress. Pandemic fear was measured using a single item on a 5-point scale ranging from 1 (not afraid at all) to 5 (very afraid). Perceived risk was assessed using a single comparative item asking respondents whether COVID-19 was less frightening than seasonal influenza. The item was reverse-coded so that higher values indicated greater perceived risk associated with COVID-19 relative to seasonal influenza. Perceived stress was measured using two items on a 5-point Likert scale. The items were averaged to form a composite measure, with higher values indicating greater stress (Cronbach’s *α* = 0.84).

#### 3.2.4 Institutional factors

Four institutional variables were included. Government performance was measured using a single 5-point item assessing respondents’ evaluation of the central government’s response to COVID-19. Institutional trust was measured using two items assessing respondents’ perceptions of community support for central and local government institutions (Cronbach’s *α* = 0.71). Police effectiveness assessed perceived police effectiveness to maintain community safety and provide public assistance. It was measured using four items averaged into a composite score (Cronbach’s *α* = 0.80). Police presence was assessed using a single item comparing the perceived frequency of community police patrols during the pandemic with the pre-pandemic period.

#### 3.2.5 Social influence factors

Two social influence variables were included. Community adherence measured respondents’ perceptions of compliance with pandemic-related regulations among people in their community, while friend adherence captured perceived compliance among their friends. Both variables were measured using single items on a 5-point scale, with higher values indicating greater perceived adherence.

Several context-specific perceptions—including pandemic fear, comparative risk, government performance, police presence, community adherence, and friend adherence—were assessed using single items designed to capture narrowly defined judgments within the institutional and policy context of COVID-19 containment in China.

### 3.3 Descriptive statistics

Table 1 presents descriptive statistics for the study variables by residential context. The full sample consisted predominantly of women, respondents aged between 22 and 39 years, and individuals with relatively high levels of education. Full-time employment was also common. For the comparative analyses, the 985 respondents were classified into rural (*n* = 88), town (*n* = 313), and urban (*n* = 584) residential groups. As explained in Section 3.1, these unequal group sizes resulted from the non-probability recruitment process rather than a predetermined sampling allocation.

**Table 1:** Descriptive statistics by residential context.

Variables	Rural (*n* = 88)		Town (*n* = 313)		Urban (*n* = 584)	
	Min–Max	Mean ± SD	Min–Max	Mean ± SD	Min–Max	Mean ± SD
Dependent variable						
Compliance willingness	2.5–5	4.67 ± 0.57	2.5–5	4.51 ± 0.54	1.5–5	4.48 ± 0.64
Demographic variables						
Gender	0–1	0.76 ± 0.43	0–1	0.66 ± 0.47	0–1	0.59 ± 0.49
Age	1–5	1.91 ± 0.91	1–6	2.39 ± 1.10	1–6	2.46 ± 0.95
Education	1–5	3.48 ± 1.10	1–5	3.55 ± 0.91	1–5	3.85 ± 0.82
Marital status	0–1	0.25 ± 0.44	0–1	0.51 ± 0.50	0–1	0.59 ± 0.49
Employment status	0–1	0.24 ± 0.43	0–1	0.56 ± 0.50	0–1	0.68 ± 0.47
Psychological factors						
Pandemic fear	1–4	2.83 ± 0.82	1–4	2.79 ± 0.81	1–4	2.86 ± 0.81
Perceived stress	1.5–5	3.68 ± 0.78	1–5	3.67 ± 0.93	1–5	3.73 ± 0.79
Perceived risk	1–5	4.10 ± 1.10	1–5	4.19 ± 0.96	1–5	4.21 ± 0.93
Institutional factors						
Government performance	3–5	4.64 ± 0.53	2–5	4.64 ± 0.57	2–5	4.65 ± 0.54
Institutional trust	3–5	4.51 ± 0.53	2.5–5	4.47 ± 0.51	2.5–5	4.51 ± 0.52
Police effectiveness	2.25–5	4.05 ± 0.65	1.75–5	4.10 ± 0.62	1.75–5	4.12 ± 0.61
Police presence	1–5	3.00 ± 1.09	1–5	3.23 ± 1.14	1–5	3.39 ± 1.09
Social influence factors						
Community adherence	3–5	4.48 ± 0.59	2–5	4.44 ± 0.56	1–5	4.48 ± 0.57
Friend adherence	3–5	4.49 ± 0.55	2–5	4.45 ± 0.59	2–5	4.49 ± 0.57

SD denotes standard deviation.

### 3.4 Analytical strategy

All analyses were conducted using Stata/MP 17.0. Statistical tests were two-sided, with *p* < 0.05 considered statistically significant. The analysis proceeded in three stages.

First, differences in compliance willingness across rural, town, and urban residential contexts were assessed using the Kruskal–Wallis test. When the omnibus test was significant, Dunn’s *post hoc* comparisons with Holm adjustment were used to identify the residential groups between which the differences occurred (57, 58). These analyses were used to test H1 while considering statistical significance alongside the absolute magnitude of the observed mean differences.

Second, hierarchical block-entry OLS regression models were estimated separately for the rural, town, and urban subsamples. Predictors were entered sequentially, beginning with demographic controls and followed by psychological, institutional, and social blocks corresponding to threat appraisal, institutional evaluation, and social influence, respectively. Changes in *R*^2^ and the associated block-level tests were used to assess the additional explanatory contribution of each set of predictors within each residential context. The subgroup model is specified in Equation (1):


$$Y_{\mathrm{ij}}=\alpha_{j}+\beta_{1j}\mathrm{DE}M_{\mathrm{ij}}+\beta_{2j}\mathrm{PS}Y_{\mathrm{ij}}+\beta_{3j}\text{IN}S_{\mathrm{ij}}+\beta_{4j}\mathrm{SO}C_{\mathrm{ij}}+\varepsilon_{\mathrm{ij}},$$ (1)

where *i* denotes the individual respondent and *j* denotes the residential context (rural, town, or urban). $Y_{\mathrm{ij}}$ represents compliance willingness, while $\mathrm{DE}M_{\mathrm{ij}}$ , $\mathrm{PS}Y_{\mathrm{ij}}$ , $\text{IN}S_{\mathrm{ij}}$ , and $\mathrm{SO}C_{\mathrm{ij}}$ denote vectors of demographic, psychological, institutional, and social predictors, respectively. The corresponding β terms represent context-specific coefficient vectors, and $\varepsilon_{\mathrm{ij}}$ captures unexplained individual-level variation. Because a coefficient being significant in one subgroup but not in another does not necessarily indicate a statistically significant difference between the coefficients, the subgroup models were used to describe within-context association patterns rather than to formally establish between-context differences.

Third, a pooled OLS model containing residence-by-predictor interaction terms for the psychological, institutional, and social factors was estimated to formally test H2. The pooled interaction model is specified in Equation (2):


$$Y_{i}=\alpha +\gamma R_{i}+\beta_{1}\;\mathrm{DE}M_{i}+\beta_{2}\mathrm{PS}Y_{i}+\beta_{3}\text{IN}S_{i}+\beta_{4}\mathrm{SO}C_{i}+$$

$$\delta_{1}(R_{i}\times \mathrm{PS}Y_{i})+\delta_{2}(R_{i}\times \text{IN}S_{i})+\delta_{3}(R_{i}\times \mathrm{SO}C_{i})+\varepsilon_{i},$$ (2)

where $R_{i}$ represents residential context, with urban areas serving as the reference category. The interaction terms $R_{i}\times \mathrm{PS}Y_{i}$ , $R_{i}\times \text{IN}S_{i}$ , and $R_{i}\times \mathrm{SO}C_{i}$ capture between-context differences in the associations of psychological, institutional, and social factors with compliance willingness. Demographic variables were included as controls but were not interacted with residential context because H2 concerns the three focal mechanisms. Joint Wald tests were used to evaluate the psychological, institutional, and social interaction terms separately, as well as all focal interaction terms collectively. When a block-level test was significant, predictor-specific Wald tests were subsequently conducted within that block. For predictors showing significant between-context heterogeneity, group-specific marginal slopes were estimated, followed by Bonferroni-adjusted pairwise comparisons. Huber–White robust standard errors were used in all OLS models to account for heteroscedasticity (59), and multicollinearity was assessed using variance inflation factors (60). Potentially influential observations were examined using Cook’s distance.

## 4 Results

### 4.1 Differences in compliance willingness across residential contexts

Public willingness to comply differed significantly across rural, town, and urban residential contexts. The Kruskal–Wallis test indicated a significant overall group difference (*p* = 0.007; *p* with ties = 0.004), suggesting that respondents’ willingness to comply with COVID-19 containment measures was not uniform across residential areas. Dunn’s *post hoc* pairwise comparisons showed that compliance willingness differed significantly between rural and town respondents (*p* = 0.001) and between rural and urban respondents (*p* = 0.001), whereas no significant difference was observed between town and urban respondents (*p* = 0.429). In both significant comparisons, rural respondents reported higher compliance willingness than the other two groups. However, the absolute mean differences were modest—0.16 points between rural and town respondents and 0.19 points between rural and urban respondents on the five-point scale. H1 was therefore supported statistically, although the substantive magnitude of the differences was limited.

**Figure 2:**
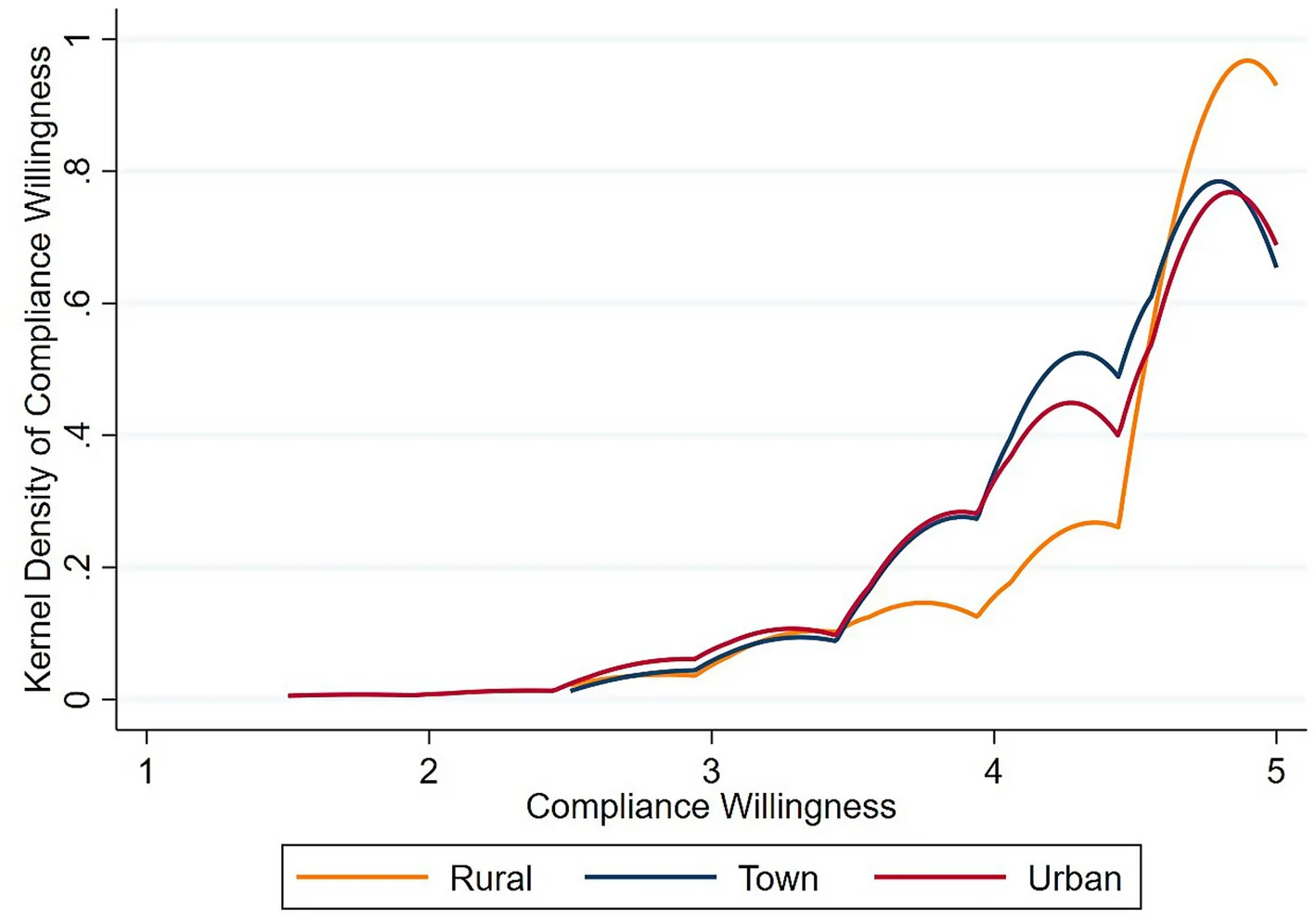
Kernel density of compliance willingness across residential contexts.

Figure 2 illustrates these group differences by presenting kernel density estimates of compliance willingness across residential contexts (61). As a smoothed representation of the distribution of compliance scores, the figure shows that all three groups were concentrated toward the upper end of the scale, indicating generally high willingness to comply across residential areas. However, the rural distribution exhibited the strongest concentration at high values, whereas the town and urban distributions were more dispersed and closely aligned with each other. These visual patterns are consistent with H1, indicating that compliance willingness differed across rural, town, and urban residential contexts.

### 4.2 Context-specific factors associated with compliance willingness

**Figure 3:**
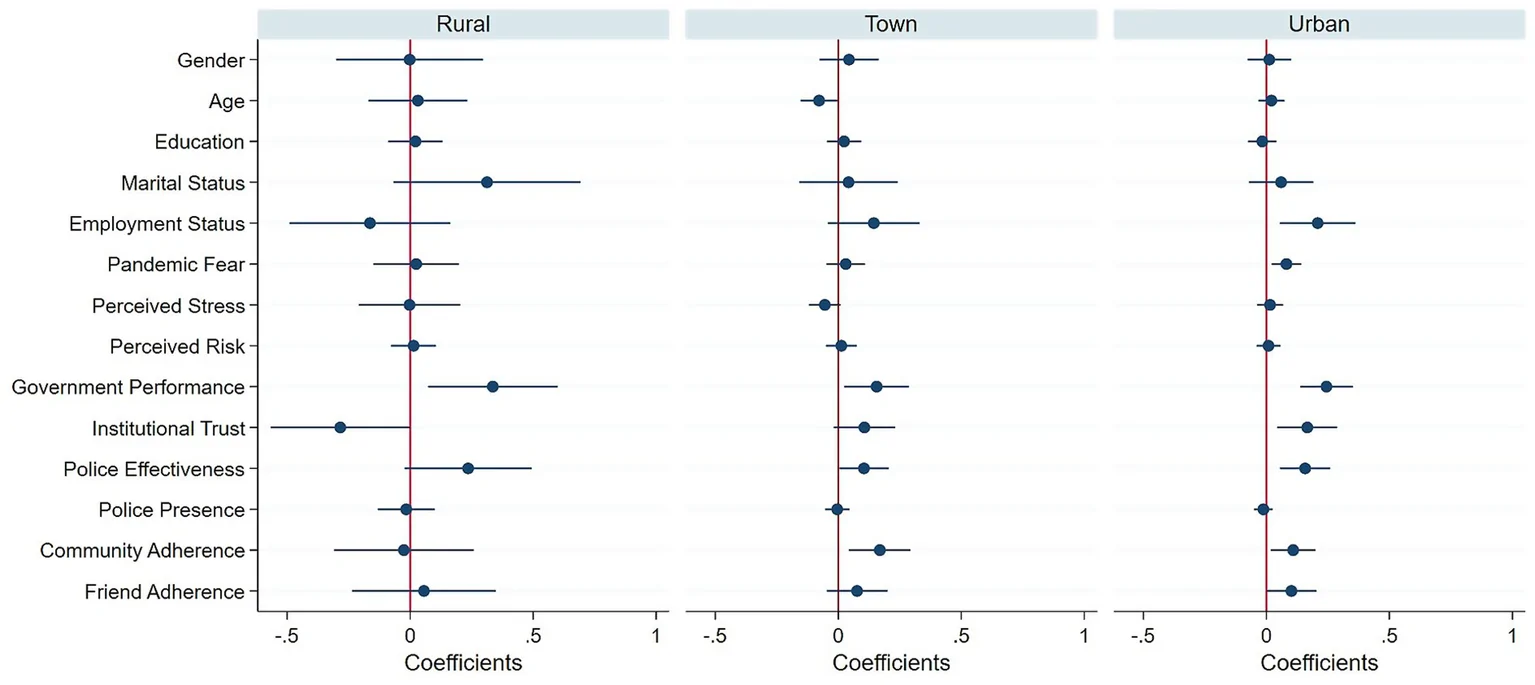
Regression coefficients across residential areas.

Figure 3 and Table 2 present the subgroup hierarchical block-entry OLS regression results for rural, town, and urban respondents. The models revealed different within-context patterns of association. Institutional factors accounted for the largest increase in explained variance in all three subsamples, while the relevance of demographic, psychological, and social factors varied across residential settings. Government performance was the only predictor positively associated with compliance willingness at the 5% level in all three models. Because differences in significance across subgroup models do not necessarily indicate statistically significant differences between coefficients, the results in this section describe within-context patterns; formal between-context comparisons are presented in Section 4.3.

**Table 2:** Subgroup hierarchical block-entry OLS regression results.

Blocks	Variables	Rural (*n* = 88)			Town (*n* = 313)			Urban (*n* = 584)		
		Coefficient	∆*R*^2^	Pr > F	Coefficient	∆*R*^2^	Pr > F	Coefficient	∆*R*^2^	Pr > F
Block 1	Gender	−0.002 (0.150)	0.053	0.103	0.044 (0.061)	0.033	0.058^*^	0.012 (0.045)	0.079	0.000^***^
	Age	0.031 (0.101)			−0.078^**^ (0.038)			0.021 (0.027)		
	Education	0.021 (0.056)			0.024 (0.036)			−0.017 (0.030)		
	Marital status	0.313 (0.191)			0.042 (0.102)			0.060 (0.067)		
	Employment status	−0.163 (0.164)			0.144 (0.095)			0.209^***^ (0.078)		
Block 2	Pandemic fear	0.024 (0.087)	0.011	0.853	0.030 (0.040)	0.023	0.111	0.082^***^ (0.030)	0.024	0.011^**^
	Perceived stress	−0.003 (0.104)			−0.055^*^ (0.033)			0.015 (0.027)		
	Perceived risk	0.014 (0.046)			0.012 (0.032)			0.009 (0.025)		
Block 3	Government performance	0.336^**^ (0.132)	0.130	0.001^***^	0.156^**^ (0.067)	0.120	0.000^***^	0.245^***^ (0.055)	0.185	0.000^***^
	Institutional trust	−0.284^**^ (0.142)			0.106^*^ (0.064)			0.166^***^ (0.062)		
	Police effectiveness	0.236^*^ (0.130)			0.104^**^ (0.051)			0.158^***^ (0.052)		
	Police presence	−0.016 (0.058)			−0.004 (0.025)			−0.012 (0.019)		
Block 4	Community Adherence	−0.025 (0.142)	0.002	0.929	0.169^***^ (0.064)	0.041	0.003^***^	0.109^**^ (0.046)	0.020	0.003^***^
	Friend Adherence	0.056 (0.147)			0.077 (0.063)			0.102^*^ (0.052)		
	Constant	3.062^***^ (0.941)			1.843^***^ (0.428)			0.543 (0.350)		
Overall			0.195	0.096^*^		0.217	0.000^***^		0.308	0.000^***^

Robust standard errors are reported in parentheses, ^***^p < 0.01, ^**^p < 0.05, ^*^p < 0.1. ∆*R*^2^ represents the change in *R*^2^.

The block-entry results further demonstrate the relative explanatory contribution of the four sets of predictors. In the rural subsample, the final model explained 19.5% of the variance in compliance willingness but was only marginal at the 10% level (*p* = 0.096). The institutional block was the only block that significantly increased explanatory power (∆*R*^2^ = 0.130, *p* = 0.001); the demographic, psychological, and social blocks made no statistically significant incremental contributions. In the town subsample, the final model explained 21.7% of the variance (*p* < 0.001). The institutional block made the largest contribution (∆*R*^2^ = 0.120, *p* < 0.001), followed by the social block (∆*R*^2^ = 0.041, *p* = 0.003). The demographic block made a smaller, marginal contribution (∆*R*^2^ = 0.033, *p* = 0.058), whereas the psychological block was not significant. The urban model explained 30.8% of the variance (*p* < 0.001). All four blocks significantly increased explanatory power, with the institutional block again contributing the most (∆*R*^2^ = 0.185, *p* < 0.001), followed by the demographic (∆*R*^2^ = 0.079, *p* < 0.001), psychological (∆*R*^2^ = 0.024, *p* = 0.011), and social blocks (∆*R*^2^ = 0.020, *p* = 0.003).

In the rural subsample, government performance was positively associated with compliance willingness (*b* = 0.336, *p* = 0.013). Institutional trust showed a negative association at the 5% significance boundary (*b* = −0.284, *p* = 0.050, rounded), while police effectiveness showed a weaker positive association at the 10% level (*b* = 0.236, *p* = 0.074). The remaining demographic, psychological, and social predictors were not statistically significant. Thus, the rural results were concentrated primarily in the institutional domain, although the relatively small rural sample warrants cautious interpretation of individual coefficients.

In the town subsample, age was negatively associated with compliance willingness (*b* = −0.078, *p* = 0.043). Among the focal predictors, government performance (*b* = 0.156, *p* = 0.021), police effectiveness (*b* = 0.104, *p* = 0.041), and community adherence (*b* = 0.169, *p* = 0.008) were positively associated with the outcome. Community adherence had the largest positive coefficient among these statistically significant focal predictors. Stress and institutional trust showed only marginal associations at the 10% level, while the other predictors were not statistically significant. This pattern indicates that both institutional evaluations and perceived community behavior were relevant to compliance willingness among town respondents.

The urban model contained the broadest range of statistically significant predictors. Employed respondents reported higher compliance willingness than those who were not employed (*b* = 0.209, *p* = 0.008), while pandemic fear was positively associated with the outcome (*b* = 0.082, *p* = 0.008). Government performance (*b* = 0.245, *p* < 0.001), institutional trust (*b* = 0.166, *p* = 0.007), and police effectiveness (*b* = 0.158, *p* = 0.003) were also positively associated with compliance willingness. Among the social factors, community adherence was significant (*b* = 0.109, *p* = 0.019), whereas friend adherence showed only a marginal association (*b* = 0.102, *p* = 0.051). Government performance had the largest coefficient among the statistically significant focal predictors in the urban model. By contrast, education, stress, perceived risk, and police presence were not significantly associated with compliance willingness in the urban model.

Diagnostic results indicated no problematic multicollinearity: all variance inflation factors were below 5, with mean VIF values of 1.88, 1.66, and 1.42 in the rural, town, and urban models, respectively. Although several observations exceeded the conservative 4/N screening threshold for Cook’s distance, all Cook’s distance values were well below 1. The maximum values were 0.165, 0.096, and 0.098, respectively, indicating that no single observation exerted extreme influence on the subgroup estimates. Overall, the subgroup models revealed distinct within-context association patterns, while the pooled interaction tests reported in Section 4.3 provide the formal assessment of H2.

### 4.3 Residential differences in predictor associations

To formally assess whether the predictor associations differed across residential contexts, a pooled OLS model was estimated with the full set of residence-by-predictor interaction terms. The joint Wald test of all 18 focal interaction terms was statistically significant, *F*(18, 950) = 1.66, *p* = 0.041, indicating that the associations collectively varied across residential contexts. Block-specific tests showed significant heterogeneity in the institutional associations, *F*(8, 950) = 2.03, *p* = 0.040, but not in the psychological, *F*(6, 950) = 0.75, *p* = 0.606, or social associations, *F*(4, 950) = 0.39, *p* = 0.814.

Further tests of the individual institutional predictors showed that institutional trust was the only variable whose association differed significantly across residential contexts, *F*(2, 950) = 4.40, *p* = 0.013. No significant between-context differences were found for government performance, *F*(2, 950) = 1.14, *p* = 0.321, police effectiveness, *F*(2, 950) = 1.60, *p* = 0.203, or police presence, *F*(2, 950) = 0.00, *p* = 0.999. Thus, although government performance and police effectiveness were associated with compliance willingness in several subgroup models, their coefficients did not differ significantly across residential contexts.

The marginal slope of institutional trust was negative in the rural subsample (*b* = −0.292, SE = 0.141, *p* = 0.038, 95% CI [−0.568, −0.016]), weakly positive in the town subsample (*b* = 0.119, SE = 0.064, *p* = 0.063, 95% CI [−0.007, 0.245]), and positive in the urban subsample (*b* = 0.161, SE = 0.062, *p* = 0.010, 95% CI [0.039, 0.283]). Bonferroni-adjusted pairwise comparisons indicated that the rural slope differed significantly from both the town slope (Δ*b* = 0.412, *p* = 0.023) and the urban slope (Δ*b* = 0.453, *p* = 0.010), whereas the town and urban slopes did not differ (Δ*b* = 0.041, *p* = 1.000). These results provide qualified support for H2: contextual heterogeneity was evident overall, but it was concentrated in the institutional domain and specifically in the association between institutional trust and compliance willingness.

## 5 Discussion

### 5.1 Interpreting heterogeneity in compliance willingness across residential contexts

The findings support H1 and provide qualified support for H2. Compliance willingness differed across rural, town, and urban settings, with rural respondents reporting higher willingness than town and urban respondents. Although the subgroup models revealed different within-context patterns, the pooled interaction analysis showed that formal between-context heterogeneity was concentrated in the institutional domain and specifically in the association between institutional trust and compliance willingness. Residential context therefore mattered, but its moderating role was more selective than the subgroup results alone might suggest.

Government performance was the most consistent institutional correlate of compliance willingness. It was positively associated with the outcome in all three subgroup models, while its interaction with residential context was not significant. This finding is consistent with studies emphasizing policy legitimacy, confidence in public authorities, and perceived governmental competence as foundations of pandemic-related compliance. Favorable evaluations of government performance may strengthen confidence that containment policies are appropriate and competently implemented, thereby increasing willingness to follow them. Its stability across residential contexts suggests that practical evaluations of governance provide a common foundation for willingness to comply. This common pattern indicates that residential context does not condition every institutional association.

Institutional trust, by contrast, exhibited a context-dependent pattern. Its association with compliance willingness was negative in rural areas, weakly positive in town areas, and positive in urban areas, with the rural slope differing significantly from both the town and urban slopes. This pattern is consistent with the institutional-environment dimension of the framework developed in Section 2.2. In urban settings, where residents may interact more frequently with formal institutions and standardized public services, institutional trust may reduce uncertainty and strengthen acceptance of official requirements. Rural willingness may instead depend more strongly on direct assessments of government performance, interpersonal relationships, and community-level implementation than on generalized institutional confidence. The similarity between the town and urban slopes suggests that towns may share some features of more formalized institutional environments. These interpretations remain tentative because the underlying contextual mechanisms were not directly measured and the rural subsample was relatively small.

The subgroup models provide descriptive insight into how the three mechanisms may operate within each setting. Rural results were concentrated primarily in the institutional domain, whereas the town model combined institutional evaluations with community adherence. This pattern is consistent with the conceptualization of towns as a hybrid context in which formal institutional presence coexists with comparatively visible community relationships. Perceived community adherence may operate as a descriptive social norm by signaling that compliance is both common and expected. The urban model contained a broader range of significant associations, including pandemic fear, which may heighten threat salience in settings characterized by greater density and mobility. However, the larger urban sample permitted more precise estimation, and differences in the number of significant predictors do not themselves demonstrate formal between-context heterogeneity. These patterns should therefore be treated as theoretically informed interpretations rather than directly tested causal mechanisms.

Turning to the overall differences addressed by H1, the higher compliance willingness among rural respondents differs from studies linking rural residence to weaker uptake of preventive practices or more limited access to health information and services. This difference may partly reflect the distinction between stated willingness and observed behavior, as well as China’s pandemic-governance context, in which grassroots mobilization, close community ties, and visible local implementation may have reinforced willingness to comply. The town findings further illustrate the analytical value of moving beyond a simple rural–urban dichotomy. Although town and urban respondents reported similar overall levels of compliance willingness, the town subgroup displayed a descriptive combination of institutional and community associations that would have been obscured if towns had been merged with either rural or urban areas. This pattern does not establish that towns differ significantly from urban areas across all mechanisms, but it supports retaining towns as a separate analytical category rather than assuming their equivalence in advance.

The timing of the survey should also be considered. Conducted in November 2022, immediately before the major relaxation of China’s pandemic-control policies in December 2022, the survey captures public attitudes during the final stage of the stringent containment period. This context may partly account for the generally high level of compliance willingness and the salience of institutional evaluations.

Finally, the non-significant findings should not be interpreted as evidence that the corresponding factors were generally unimportant. Perceived risk and police presence showed no stable independent associations, while the psychological and social blocks contributed little explanatory power in the rural model. The high and compressed distribution of compliance willingness may have limited the variation available to explain, and the simultaneous inclusion of related predictors may have reduced their independent contributions. China’s stringent and coordinated pandemic response may also have made institutional evaluations more salient than some individual perceptions. These explanations remain provisional, particularly given the lower precision of the rural estimates.

### 5.2 Public health policy implications

The findings suggest that strategies intended to strengthen willingness to comply should combine credible governance with context-sensitive implementation. Across residential settings, authorities should strengthen confidence through transparent policy rationales, consistent communication, responsive services, and visible implementation capacity. The stable positive association between government performance and compliance willingness suggests that effective policy delivery is more broadly relevant than persuasive messaging alone.

Implementation should nevertheless reflect local conditions. In rural areas, information can be delivered through trusted local channels, including village committees and primary healthcare workers, with online communication supplemented by direct outreach where digital access is limited. Given the distinct rural pattern involving institutional trust, confidence is more likely to be strengthened through accessible services, procedural transparency, and demonstrable responsiveness than through abstract appeals to institutional authority. Town areas require a somewhat different combination of formal communication and community engagement. Local authorities can work with neighborhood organizations, healthcare providers, and resident groups to explain service arrangements, address practical concerns, and make collective adherence visible. Such an approach may connect institutional credibility with supportive social norms without relying primarily on coercive messaging.

Urban strategies should coordinate communication across community platforms, workplaces, healthcare institutions, and digital channels. Messages should communicate risk clearly without unnecessarily amplifying fear and should account for differences in employment and mobility. Community-level information can also make supportive behavioral norms more visible within socially diverse urban populations. These distinctions do not imply that each residential context requires an entirely separate policy system. Rather, a common foundation of credible performance and accessible communication should be adapted through locally appropriate channels and forms of engagement.

### 5.3 Limitations and future research

Several limitations should be considered when interpreting the findings. First, the cross-sectional design does not permit causal inference, and the observed relationships should therefore be understood as associations. Reliance on self-reported data may introduce common method bias and encourage socially desirable responses, particularly under strict pandemic policies. The dependent variable captured stated willingness rather than observed behavior and did not encompass the full range of preventive practices. The concentration of compliance willingness toward the upper end of the scale may have reduced the variation available to explain, while the use of single-item predictors may have reduced measurement precision. Future studies could extend the present analysis through longitudinal designs, behavioral measures, and more comprehensive instruments.

Second, the non-probability online sample was not intended to represent the regional population. The sample consisted predominantly of women, younger respondents, and individuals with relatively high levels of education, while the relatively small rural subsample may have reduced the precision of the rural estimates. These sample characteristics should be considered when generalizing the findings beyond the surveyed areas. Nevertheless, the inclusion of rural, town, and urban respondents provides a useful basis for identifying residential variation within a shared national institutional setting. Future research could assess the broader applicability of these patterns using probability-based and more balanced samples across regions of China, as well as comparative studies in other institutional contexts.

Finally, the models could not incorporate all potentially relevant contextual conditions, such as local public health capacity, economic constraints, policy intensity, and implementation practices. These factors may help explain why particular associations vary across residential settings. Future multilevel research could link individual responses with community- and regional-level indicators to examine these contextual influences more directly.

## 6 Conclusion

This study demonstrates that compliance willingness and its correlates vary across residential contexts. Rural respondents reported higher willingness than town and urban respondents, while government performance was a stable positive correlate across all three settings. Formal interaction tests showed that contextual heterogeneity was concentrated in institutional trust, whose association with compliance willingness differed between rural areas and the other two settings. By treating towns as a distinct analytical category, the study moves beyond the conventional rural–urban divide and shows that towns combine institutional and community characteristics that may otherwise be overlooked. The findings further suggest that residential context conditions how institutional evaluations relate to preventive intentions. Accordingly, public health strategies seeking to strengthen willingness to comply may benefit from maintaining a common foundation of credible governance while adapting communication and community engagement to local conditions. Future research should examine these patterns using cross-regional and cross-national comparisons, longitudinal or behavioral data, and multilevel models incorporating health-system and policy-implementation indicators.

## Data Availability

The raw data supporting the conclusions of this article will be made available by the authors, without undue reservation.

## Footnotes

1 The sample covers the following cities: Beijing (Beijing Municipality); Shijiazhuang, Tangshan, Qinhuangdao, Langfang, Zhangjiakou, Baoding, Chengde, Xingtai, Cangzhou, Handan, and Hengshui (Hebei Province); and Shenyang, Dalian, Dandong, Jinzhou, Fushun, Fuxin, Anshan, Yingkou, Tieling, Panjin, Benxi, Chaoyang, Liaoyang, and Huludao (Liaoning Province).

## Appendix

**Table A1:** Questionnaire items.

Construct	Item
Dependent variable	
Compliance willingness	I am willing to comply with COVID-19 containment regulations.
	I am willing to cooperate with the police regarding COVID-19 containment regulations.
Independent variables	
Psychological factors	
Pandemic fear	I am afraid of becoming infected with COVID-19.
Perceived stress	I feel stressed.
	I feel anxious.
Perceived risk	COVID-19 is less frightening than seasonal influenza.
Institutional factors	
Government performance	The central government has performed well in responding to the COVID-19 pandemic.
Institutional trust	People in my community support national/central government institutions.
	People in my community support local government institutions.
Police effectiveness	The police are effective in handling community safety issues.
	The police respond quickly to emergency calls or requests for assistance.
	The police are effective in preventing crime.
	The police are effective in assisting victims.
Police presence	Police patrols in my community during the COVID-19 pandemic are as frequent as before the pandemic.
Social influence factors	
Community adherence	Most people in my community comply with the pandemic-related regulations.
Friend adherence	Most of my friends comply with the pandemic-related regulations.
